# Positive Synergistic Effects of Quercetin and Rice Bran on Human Gut Microbiota Reduces *Enterobacteriaceae* Family Abundance and Elevates Propionate in a Bioreactor Model

**DOI:** 10.3389/fmicb.2021.751225

**Published:** 2021-09-30

**Authors:** Sudeep Ghimire, Supapit Wongkuna, Ranjini Sankaranarayanan, Elizabeth P. Ryan, G. Jayarama Bhat, Joy Scaria

**Affiliations:** ^1^Department of Veterinary and Biomedical Sciences, South Dakota State University, Brookings, SD, United States; ^2^South Dakota Center for Biologics Research and Commercialization, Brookings, SD, United States; ^3^Department of Pharmaceutical Sciences, South Dakota State University, Brookings, SD, United States; ^4^Department of Environmental and Radiological Health Sciences, Colorado State University, Fort Collins, CO, United States

**Keywords:** gut microbiome, bioreactor, metagenomics, flavonoid, quercetin, rice bran, prebiotic

## Abstract

Dietary fiber and flavonoids have substantial influence on the human gut microbiota composition that significantly impact health. Recent studies with dietary supplements such as quercetin and rice bran have shown beneficial impacts on the host alongside a positive influence of the gut microbiota. The specific bacterial species impacted by quercetin or rice bran in the diet is not well understood. In this study, we used a minibioreactor array system as a model to determine the effect of quercetin and rice bran individually, as well as in combination, on gut microbiota without the confounding host factors. We found that rice bran exerts higher shift in gut microbiome composition when compared to quercetin. At the species level, *Acidaminococcus intestini* was the only significantly enriched taxa when quercetin was supplemented, while 15 species were enriched in rice bran supplementation and 13 were enriched when quercetin and rice bran were supplemented in combination. When comparing the short chain fatty acid production, quercetin supplementation increased isobutyrate production while propionate dominated the quercetin and rice bran combined group. Higher levels of propionate were highly correlated to the lower abundance of the potentially pathogenic *Enterobacteriaceae* family. These findings suggest that the combination of quercetin and rice bran serve to enrich beneficial bacteria and reduce potential opportunistic pathogens. *In vivo* studies are necessary to determine how this synergy of quercetin and rice bran on microbiota impact host health.

## Introduction

Gut microbiome influences health and disease (Gentile and Weir, 2018; Maji et al., 2018; Pulikkan et al., 2018) and is highly affected by diet (Turnbaugh et al., 2009; David et al., 2014; Dhakan et al., 2019). Some dietary components are known to enhance host health by promoting the growth of beneficial bacteria in the gut, termed as “prebiotics” (Gibson and Roberfroid, 1995; Slavin, 2013). Quercetin and rice bran have been used as prebiotics separately in mice and pig models, showing significant health benefits to the host (Henderson et al., 2012; Goodyear et al., 2015; Lin et al., 2019). Rice bran, a byproduct of the rice milling process, is available, affordable, sustainable, and a globally produced source of prebiotics. It is known to supplement nutrients (Zarei et al., 2017), increase beneficial bacteria growth, enhance gut mucosal immunity (Henderson et al., 2012), and prevent diseases (Kumar et al., 2012; Goodyear et al., 2015; Lei et al., 2016; Islam et al., 2017). Similarly, quercetin (3,3′,4′,5,7-pentahydroxyflavone) represents an important subgroup of flavonoids found in fruits and leafy vegetables (D’Archivio et al., 2007). Quercetin (QC) has received substantial attention in the past few years from the scientific community for exerting anti-inflammatory effects (Comalada et al., 2005; Stewart et al., 2008) and its potential health-promoting properties in the treatment or prevention of cardiovascular diseases (Egert et al., 2009), lung and colorectal cancers (Linsalata et al., 2010; Darband et al., 2018), and colitis (Hong and Piao, 2018; Lin et al., 2019). Furthermore, the mammalian body can endure high levels of quercetin without any significant adverse health effects (Harwood et al., 2007).

Despite multiple studies corroborating the beneficial effect of quercetin and rice bran on the host, their effect on microbiota varies significantly from study to study and has lower taxonomic resolution (Sheflin et al., 2015; Tamura et al., 2017; Hong and Piao, 2018; Zambrana et al., 2019). Inter-individual variation in *in vivo* studies are often due to multiple host-related factors which makes it difficult to interpret microbiome results (Zoetendal et al., 1998). In contrast, studying the effect of dietary ingredients on the microbiome in the absence of confounding host factors can help to better understand the complex microbial interactions. Bioreactors have been used as model systems to study how dietary ingredients shape microbiomes (Chung et al., 2019). The use of bioreactors allows precise control of the environmental conditions that affect microbiome composition which provide increased reproducibility and reveal microbial interactions in a more defined way. Minibioreactor array is an *in vitro* anaerobic model system that simulates hindgut conditions for growth of complex, stable microbiota without interference of host factors (Auchtung et al., 2015). Furthermore, this system helps to identify microbial biotransformations and allows for measuring metabolites produced (National Academies of Science, Engineering, and Medicine, 2017). We used minibioreactor array systems to gain deeper understanding of how the microbiota responds to quercetin and/or rice bran without host interference, and hypothesized that the combination of quercetin and rice bran will have a synergistic positive effect on the gut microbiota and microbiota metabolism. We show that combined supplementation of quercetin and rice bran in the minibioreactors model for human gut microbiome significantly reduced members of *Enterobacteriaceae* family and resulted in higher propionate production. This study provides novel insights to species-level shifts in the human gut microbiome in the presence of quercetin and/or rice bran supplementation.

## Materials and Methods

### Donor Samples, Mini-Bioreactor Array Preparation and Sample Collection

We obtained fresh fecal samples from six healthy donors with no prior history of antibiotic consumption in the past year. The pooled fecal sample from six individuals was used as the inoculum as our prior study showed that pooled sample represented the individual microbial composition (Ghimire et al., 2020).

**FIGURE 1:**
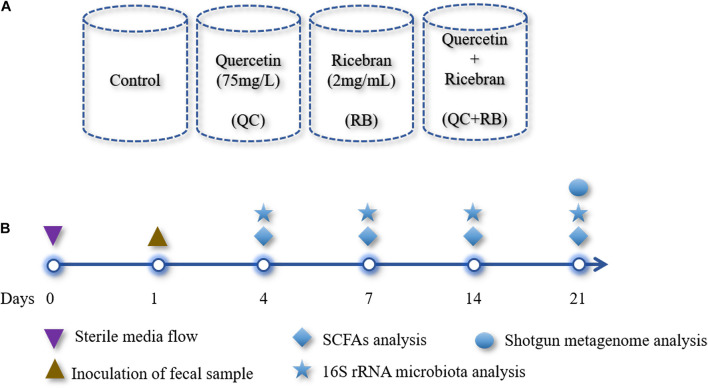
Overview of the study design: **(A)** A schematic diagram shown the design of four different media conditions used in this study. Single substrates (quercetin or rice bran) or the mixture of quercetin and rice bran were used at the final concentration of 75 and 2 mg/mL as shown above in the base medium BHI (see section “Materials and Methods”). Final pH of the media was adjusted to 6.8 ± 0.2 for all conditions. Each condition was run in triplicate and were inoculated with the same fecal inoculum (see section “Materials and Methods”). **(B)** Outline of the bioreactor experiment showing time points for fecal inoculation and sample collection for SCFAs and microbial community analysis.

The modified BHI medium (Ghimire et al., 2020) was used as a control medium. Heat-stabilized rice bran (RBT 300) was purchased from Rice Bran Technologies (Sacramento, CA, United States), and the extract was prepared as described previously (Kumar et al., 2012) and then added to the control medium (final concentration: 2 mg/mL): designated as RB. Quercetin was added to the media at a final concentration of 75 mg/L and is referred to as QC. The final experimental group consisted of modified BHI medium with the additions of quercetin (75 mg/L) and rice bran extract (2 mg/mL) and is identified as QC + RB. Mini-bioreactors (MBRAs) were sterilized, assembled and the experiment was performed as described previously (Auchtung et al., 2016) with minor modifications. The total experimental volume in each mini-bioreactor was set at 15 ml. To maintain anaerobic conditions, the bioreactors were placed inside an anaerobic chamber (Coy Laboratories) containing with 85% nitrogen, 10% carbon dioxide, and 5% hydrogen. The temperature was maintained at 37°C throughout the experiment. Initially, pH of each of the medium for inflow into the mini-bioreactors was adjusted to 6.8 and not altered throughout the experiment. Two 24 channel Watson-Marlow pumps were used pumping media in and removing excess fermented medium from the bioreactor. For this purpose, the input and output on Watson Marlow pumps were set at 1 and 2 rpm, respectively. The rotating magnetic stirrer was set at 130 rpm. The media (Control, QC, RB, and QC + RB) (Figure 1A) were allowed to flow continuously for 24 h each in triplicate. Three hundred microliters of the inoculum was introduced into all wells with a retention time of 16 h. The continuous flow model was operated up to 21 days post-inoculation (Figure 1B). Five hundred microliters of the media was collected for sequencing at day 0 (inoculum), days 4, 7, 14, and 21 and directly frozen to −80°C. Also, samples for short chain fatty acid (SCFAs) determination were collected at days 4, 7, 14, and 21 post-inoculation with dietary treatments and controls (Figure 1B). Each media condition was run in triplicate and samples were collected at day 4, 7, 14, 21 resulting in 48 samples from four conditions (control, QC, RB, and QC + RB). Duplicate samples from the inoculum was also used for sequencing. Therefore, a total of 50 samples were used for DNA isolation and sequencing.

### Microbial DNA Extraction and Sequencing

DNA isolation was performed on 50 samples including duplicate inoculum samples. The DNA was extracted from 500 μl of the sample using a Powersoil DNA isolation kit (MoBio Laboratories Inc., CA, United States) following the manufacturer’s instructions. After extraction, the quality of DNA was measured using NanoDrop^TM^ one (Thermo Fisher Scientific, DE, United States) and quantified using Qubit Fluorometer 3.0 (Invitrogen, CA, United States). The DNA samples were stored at −20°C until further use. To analyze the variation of the microbial composition over time, all samples were amplicon sequenced using an Illumina MiSeq platform with paired-end V3 chemistry. The library was prepared using an Illumina Nextera XT library preparation kit (Illumina Inc., CA, United States) targeting V3-V4 regions of the 16S rRNA. The libraries were bead normalized and multiplexed before loading into the sequencer. We also performed shotgun metagenome sequencing on twelve samples obtained from day 21 to identify species level taxonomical differences between the experimental groups. We used the Nextera XT kit (Illumina, San Diego, CA, United States) for the preparation of the shotgun metagenome sequencing library. The library was then sequenced using paired end 300 base sequencing chemistry using a MiSeq platform.

### Data Analysis

The time-series changes in the microbial communities were analyzed using 16S rRNA community analysis in Quantitative Insights into Microbial Ecology framework (QIIME, Version 2.0) (Bolyen et al., 2019). Briefly, the demultiplexed reads obtained were quality filtered using q2- demux plugin and denoised applying DADA2 (Callahan et al., 2016). All amplicon sequence variants were aligned with *mafft* (Katoh et al., 2002) to construct a phylogeny with fasttree2 (Price et al., 2010). The outputs rooted-tree.qza, table.qza, taxonomy.qza were then imported into R (R Core Team, 2017) for analysis using *phyloseq* (McMurdie and Holmes, 2013). Shannon diversity and bray curtis dissimilarity indices were calculated as alpha and beta diversity metrices. Kruskal–Wallis test at *p* = 0.05 was performed to compare the species richness between the groups. The reads were normalized by rarefying to 30,000 reads using rarefy_even_depth function from phyloseq package in R and the taxonomy was assigned to amplicon sequence variants using the q2-feature-classifier (Bokulich et al., 2018) using Greengenes as the reference (McDonald et al., 2012). Rarefying the reads to 30,000 were enough to estimate total diversity and taxonomy (Supplementary Figure 1A). Initially, a total of 947 amplicon sequence variants were identified from 50 samples. The average number of non-chimeric reads per sample obtained in Qiime2 pipeline was 92,512 ± 27,631 (mean ± SD). The amplicon sequence variants obtained were filtered to select those which are present in at least 20% of samples with a count of 10 each or amplicon sequence variants with > 0.001% of total median count reducing the total number to 581.

For shotgun metagenomes, raw fastq sequences were quality controlled using Fastqc^1^ and host reads were removed using metaWRAP pipeline (Uritskiy et al., 2018). Filtered reads were analyzed for taxonomy using Kaiju (Menzel et al., 2016) against the proGenomes database (Mende et al., 2017) using default parameters. The percentage abundance of each taxon was plotted using Explicit v2.10.5 (Robertson et al., 2013). The samples from day 21 yielded a total of 39,332,649 reads of which 70. 86% were classified to 161 different species (species with less than 100 reads were removed), whereas 185,188 (5.55%) and 9,278,647 (23.59%) reads remained as unclassified bacteria or unassigned to non-viral species, respectively. In addition, the raw counts obtained for each taxon were used for calculation of alpha and beta diversity using the *phyloseq* package in R. For identification of differentially abundant taxa in QC, RB and QC + RB compared to control medium, the raw counts of abundance of each taxon was then Log10(x + 1) transformed and fed to DESEq2 (Love et al., 2014) package in R. Bacterial taxa which significantly altered from the control were further filtered out with the criteria of at least log2foldchange (Log2FC) of ≥ 2 and p*adj* value > 0.05. The enriched taxa were selectively analyzed for their co-relation to SCFAs phenotypic data using Spearman correlation method in R. In addition, we performed *de novo* assembly of the quality filtered reads using metaSPAdes (SPAdes 3.13.0) (Nurk et al., 2017). The assembled *fasta* sequences were annotated using Prokka (Seemann, 2014) using default parameters. The obtained amino acid *fasta* sequences were searched for the KEGG modules using GHOSTKOALA (Kanehisa et al., 2016) with default parameters. The KEGG modules thus obtained were analyzed for complete, 1 block missing, 2 blocks missing and incomplete pathways and visualized as heatmap using Morpheus web server^2^.

### Estimation of Short Chain Fatty Acids

For the estimation of SCFAs, 800 μl of samples were collected from each mini-bioreactor, mixed with 160 μl of 25% m-phosphoric acid and frozen at −80°C until further analysis. Later, the frozen samples were thawed and centrifuged (>15,000 × *g*) for 20 min. Five hundred microliters of supernatant was collected in the tubes before loading into the gas chromatography- mass spectrometry (GC-MS) (Agilent Technologies, United States) for analysis (Ghimire et al., 2020). The SCFA concentrations were compared between the groups using the Kruskal–Wallis test followed by the Dunn test with Benjamini-Hochberg correction in R and visualized using GraphPad Prism 6.0.

## Results

### Rice Bran Produced Larger Shift in Microbiota Composition When Compared to Quercetin

**FIGURE 2:**
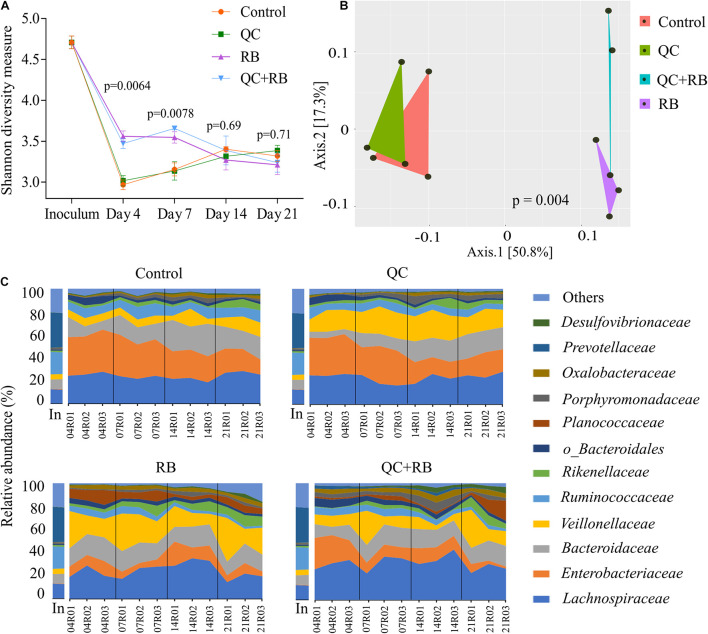
Gut microbiota compositional changes following quercetin and rice bran supplementation **(A)** Alpha diversity of four groups (control, QC, RB, and QC + RB) at day 4, 7, 14, and 21 from amplicon sequencing data. **(B)** Beta diversity among the control, QC, RB and QC + RB groups were obtained using shotgun metagenome sequence analysis on day 21. “MDS” ordination followed by Bray-Curtis dissimilarity calculation was used to visualize the differences between the groups (adonis, *p* = 0.004). **(C)** Temporal family-level composition of the most abundant 12 bacterial taxa at day 4, 7, 14, and 21 and inoculum determined using 16S rRNA community profiling for Control, QC, RB, and QC + RB conditions.

We used a minibioreactor array system to analyze the impact of QC and RB on the gut microbiota using the study design shown in Figure 1. The microbial community richness indicated by the Shannon diversity index showed significant differences in the early days (days 4 and 7) due to addition of rice bran in the media whether alone or in combination with quercetin (Figure 2A). No significant changes in the richness was observed among the groups on day 14 and day 21, suggesting the stabilization of the microbial communities by day 14. Also, on day 21, shotgun metagenome analysis showed no differences in the richness between the four conditions (Supplementary Figure 1B). In contrast, marked differences between the communities were evident as early as day 4 by beta diversity analysis (Supplementary Figure 2A). Rice bran (RB) supplementation showed a greater shift in the bacterial community as shown on days 7, 14, and 21 (Supplementary Figures 2B–D). Based on Bray-Curtis dissimilarity metrics, RB and QC + RB treatments were similar to each other and clustered separately from control and QC. This reveals that quercetin causes less shift in microbiome composition when compared to rice bran (Supplementary Figure 2). Shotgun metagenome sequencing at day 21 also indicated significant differences in the community profile, primarily influenced by the addition of rice bran extract in the medium (Figure 2B).

When examined taxonomically, the baseline inoculum composition was dominated by *Prevotellaceae* (29.9 ± 0.87%), followed by *Ruminococcaceae* (18.82 ± 0.81%) and *Lachnospiraceae* (12.66 ± 0.4%). Also, *Enterobacteriaceae* was found to be very low in inoculum (0.33 ± 0.097%). However, the abundance of *Prevotellaceae* was reduced to ∼0.0% after 21 days in all treatment groups suggesting that not all taxa in the inoculum were supported in the bioreactor model. Over time, there was a major shift in the composition of the microbiota between the groups starting as early as day 4 (Figure 2C). The control and quercetin medium were dominated by *Enterobacteriaceae*, followed by *Lachnospiraceae* at day 4. However, by day 21, *Lachnospiraceae* dominated the control and QC conditions followed by *Bacteroidaceae*. The abundance of *Enterobacteriaceae* was reduced from 34.16 ± 1.92% and 33.26 ± 1.11% on day 4 to 17.62 ± 4.2% and 18.53 ± 3.88% in control and QC medium, respectively, by day 21 (Figure 2C). Even though ∼50% reduction of abundance of *Enterobacteriaceae* was observed for both control and QC medium at day 21 compared to day 4, the population of *Enterobacteriaceae* tend to stabilize. However, *Enterobacteriaceae* were highly reduced in both RB and QC + RB conditions. In RB medium, *Enterobacteriaceae* was 10.212 ± 2.14% at day 4 which was further reduced to 6.33 ± 2.59% by day 21. At day 21, *Veillonellaceae* and *Lachnospiraceae* dominated the RB medium. Similarly, the dominant family were *Lachnospiraceae, Veillonellaceae* and *Bacteroidaceae* in the quercetin and rice bran combination (QC + RB) on day 21. Strikingly, on day 21, the abundance of *Enterobacteriaceae* was reduced by ∼18.22% to 4.20 ± 3.16% compared to day 4 on QC + RB condition. At day 21, when mean abundance of *Enterobacteriaceae* was compared between control, QC, RB and QC + RB, significant reduction was observed in RB and QC + RB (Kruskal–Wallis, *p* = 0.0064).

**FIGURE 3:**
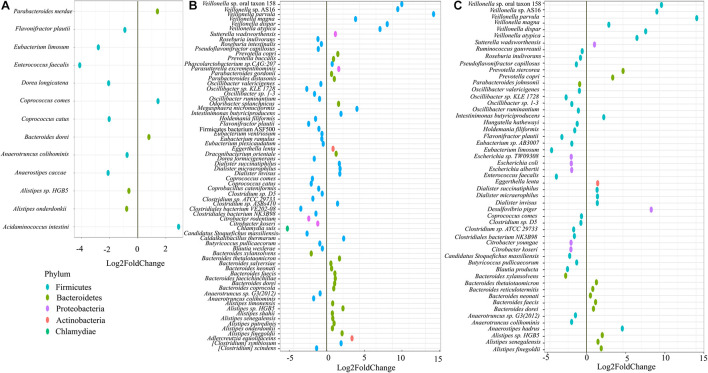
Impact of quercetin and rice bran supplementation on microbiota bacterial species. Bacterial species that are significantly altered in **(A)** quercetin (QC), **(B)** rice bran (RB), and **(C)** QC + RB when compared to the control medium. The differential significance of the species was calculated by using DESEq2 (Supplementary Table 1) from shotgun metagenome sequence analysis at day 21. The left column represents bacterial species-level taxa while the colors indicate their corresponding phyla (“Blue” = *Firmicutes*, “Brown” = *Bacteroidetes*, “Pink” = *Proteobacteria*, “Orange” = *Actinobacteria*, “Green” = *Chlamydiae*).

On day 21, we performed shotgun metagenome analysis to identify the species level differences among the four groups (Supplementary Figure 3 and Supplementary Table 1). Based on DeSEQ2 analysis, 53 species were significantly altered in QC + RB medium (Figure 3). The combination (QC + RB) enriched 13 additional species and reduced 13 others when the cut off value of Log2FC ≥ 2 and p*adj* value > 0.05 was applied (Supplementary Table 1). Specifically, abundance of *Escherichia* sp. TW09308, *E. coli*, *E. albertii*, *Citrobacter koseri*, and *C. youngae* were reduced. The *Citrobacter* species, including *C. rodentium*, were also reduced in RB medium; however, *Escherichia* species were not significantly reduced in this medium (Figure 3).

Further, 13 species were altered significantly in the quercetin group (Figure 3A), and 70 species in the rice bran group compared to control (Figure 3B). With a cut off Log2FC ≥ 2 and p*adj* value > 0.05, *Acidaminococcus intestine* was the only enriched taxa in media supplemented with quercetin while *Coprococcus catus, Dorea longicatena, Anaerostipes caccae, Eubacterium limosum*, and *Enterococcus faecalis* were highly reduced. Interestingly, the population of *Flavonifractor plautii*, a flavonoid metabolizing bacterium (Braune and Blaut, 2016; Gupta et al., 2019) was significantly reduced in quercetin supplementation. Rice bran supplementation alone enriched 15 species, including six different species from *Veillonella* (Supplementary Table 1).

To gain functional insights into the prebiotic supplementation, we performed comparative pathway analysis between controls and treatments using KEGG modules search for the metagenomic samples at day 21 (Supplementary Figure 4). Results from this analysis showed that cysteine biosynthesis (M00338), lysine biosynthesis (M00031) and ornithine biosynthesis (M00763) pathways were absent in QC when compared to control. Interestingly, lysine biosynthesis and ornithine biosynthesis pathways were retained in RB but lost in QC + RB suggesting that addition of QC leads to elimination of lysine, and ornithine biosynthesis pathways completely. Also, reductive pentose phosphate cycle (M00165 and M00166), cysteine biosynthesis (M00609), C10-C20 isoprenoid biosynthesis (M00365) and catechol meta-cleavage (M00569) were more complete in RB and QC + RB (Supplementary Figure 4). M00569 pathway is responsible for the convertion of catechol moiety found in quercetin to either acetyl-CoA or 4-methylcatechol which is finally converted to propanoyl-CoA suggesting that QC + RB medium enhances amino acids biosynthesis along with production of propionate.

### Quercetin and Rice Bran Combination Yield Higher Propionate Levels and Reduces Members of *Enterobacteriaceae* Family

**FIGURE 4:**
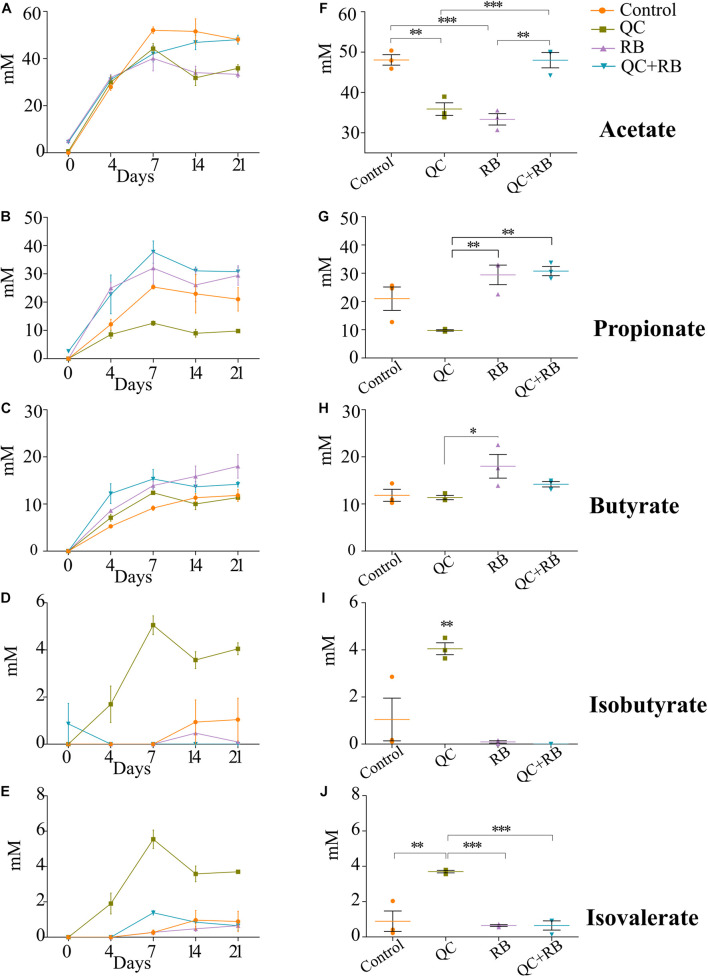
Effect of quercetin (QC) and rice bran (RB) on short Chain Fatty Acids (SCFAs) production in QC, RB, and QC + RB compared to control medium in minibioreactors. **(A–E)** Represent the periodic variation of acetate, propionate, butyrate, isobutyrate and isovalerate at day 0, 4, 7, 14, and 21, respectively. **(F–J)** Represent the comparison of concentrations of acetate, propionate, butyrate, isobutyrate and isovalerate, respectively, from control, QC, RB, and QC + RB medium at day 21 (endpoint). Kruskal–Wallis test was performed between the groups and *post hoc* (Dunn test) analysis was performed to identify the different significant groups. “*, **, and ***” represents significance at *p* < 0.05, *p* < 0.01 and *p* < 0.001 respectively. Error bars represent standard error of the mean of data obtained from three different bioreactors.

Along with the changes in the taxonomy of the microbial community, diet alters the metabolic profile of a community (Sonnenburg and Sonnenburg, 2014; Sonnenburg and Backhed, 2016). To understand how these dietary substrates have altered the fermentation potential of the microbiota, we measured short chain fatty acids (SCFAs) from each minibioreactor on days 4, 7, 14, and 21. We observed that the amount of each SCFA produced was stable by day 14 and at the endpoint, there were marked differences between the groups (Figures 4A–E). At day 21, communities formed in the control and RB weighted strongly toward acetate and butyrate production, respectively (Supplementary Figure 5). Statistically at day 21, acetate production in QC + RB group (48.01 ± 3.3 mM) was significantly different from the RB group (33.33 ± 2.44 mM) (Figure 4F). The butyrate production in the RB medium was higher but not significantly different compared to the other groups (Figure 4H). Similarly, the medium supplemented with quercetin and rice bran (QC + RB) weighted toward the production of propionate and butyrate (Supplementary Figure 5). Rice bran alone and combined quercetin and rice bran supplementation significantly raised propionate levels by at least threefold compared to the QC group alone (Figure 4G). In contrast, when QC was supplemented in the medium, it shifted the production of minor SCFAs (Figures 4I,J and Supplementary Figure 5). However, only isobutyrate production was significantly higher in QC compared to QC + RB group (Figure 4I).

**FIGURE 5:**
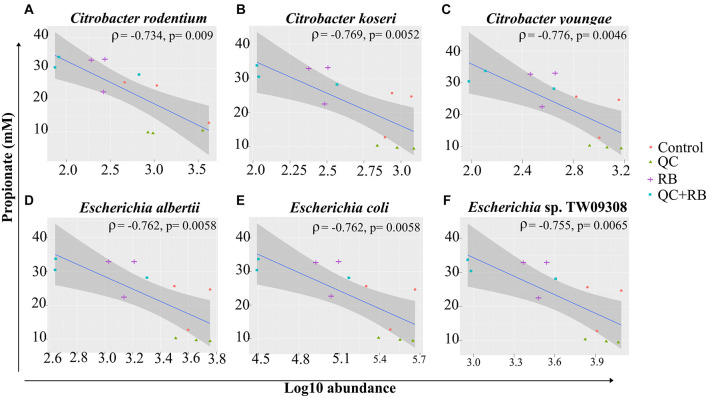
Spearman correlation of log10 abundance of the members of *Enterobacteriaceae* family **(A)** *Citrobacter rodentium*, **(B)** *Citrobacter koseri*, **(C)** *Citrobacter youngae*, **(D)** *Escherichia albertii*, **(E)** *Escherichia coli*, and **(F)** *Escherichia* sp. TW09308 at day 21 determined by shotgun metagenomics sequence analysis to levels of propionate in the medium. A negative correlation is expressed by negative values of correlation coefficient “rho” (ρ) with corresponding *p*-values.

Short chain fatty acids production by microbiota has been associated with pathogen inhibition to benefit the host (Hung et al., 2013). Physiological levels of SCFAs are reported to reduce *Enterobacteriaceae* members by pH mediated action (Sorbara et al., 2019). Specifically, propionate production by a propionate producing consortium has been shown to reduce antibiotic induced dysbiosis (El Hage et al., 2019). In this study, as higher production of propionate weighted toward QC + RB medium at day 21, we estimated the correlation of abundances of significantly altering members of *Enterobacteriaceae* to propionate levels. Significant high negative correlations were observed between the abundance of the members of *Enterobacteriaceae* family and propionate with media change (Figure 5). *Citrobacter rodentium* (ρ = −0.734, *p* = 0.009), *C. koseri* (ρ = −0.769, *p* = 0.0052), *C. youngae* (ρ = −0.776, *p* = 0.0046), *Escherichia albertii* (ρ = −0.762, *p* = 0.0058), *E. coli* (ρ = −0.762, *p* = 0.00587) and *Escherichia* sp. TW09308 (ρ = −0.755, *p* = 0.0065) (Figures 5A–F) were greatly reduced in QC + RB medium, whereas propionate levels were at least threefold and 1.5 fold higher compared to QC and control medium.

## Discussion

*In vivo* studies have shown changes in microbial communities due to quercetin or rice bran and describe the improvement of colonization resistance and reduction of colon cancer (Kumar et al., 2012; Zhang et al., 2015; Lin et al., 2019). However, these studies are marked by high variations of the microbiota composition likely due to host factors. Additionally, very little is known about the combined effect of quercetin and rice bran on gut microbiota. Thus, we focused on understanding the impact of quercetin and rice bran separately and in combination using a minibioreactor array model (Auchtung et al., 2016). Also, to gain insights into taxonomical composition, we performed 16S rRNA sequencing over time and shotgun metagenome sequencing at the endpoint.

The alpha diversity analysis indicated that the richness of the communities in bioreactors stabilized by day 14 (Figure 2A). However, the taxonomical differences were evident as early as day four following inoculation. The changes in the relative abundances of taxa after day 14 were lower in control and quercetin supplemented medium whereas rice bran and combined quercetin with rice bran conditions had a more homogenized microbial composition after day 4 (Figure 2C). This indicates that the time required for the stabilization of communities may vary with the substrate used in minibioreactors in contrast to the previously determined timeframe of 1 week following fecal inoculation (Auchtung et al., 2015). Such changes in the time required for stabilization and diversity between the groups can be attributed ecologically to the stochastic rearrangement of the microbial species and their interactions at early stages because of selection pressure by substrate (Vellend, 2010). We also found that members of dominant family such as *Prevotellaceae* in donor microbiota did not retain high abundance in the minibioreactor system. This is probably because the modified BHI medium we used do not contain enough complex carbohydrates to sustain high abundance of species in *Prevotellaceae* family.

Rice bran is a nutrient-dense food with a unique profile and ratio of bioactive phytochemicals such as gamma oryzanol, tocotrienols, ferulic acid, vitamin B, beta-sitosterol and many others (Zarei et al., 2017). It has been reported to be effective in preventing *Salmonella typhimurium* (Kumar et al., 2012; Goodyear et al., 2015; Rubinelli et al., 2017), rotavirus (Yang et al., 2015; Nealon et al., 2017) and norovirus (Lei et al., 2016) infections. However, very few *in vivo* studies have highlighted its effect on gut microbiota and the reports have striking differences. In a clinical trial, Zambrana et al. (2019) showed significant enrichment of *Veillonella*, *Megasphaera* and *Dialister* species at the genus level from gut samples of children from either Nicaragua or Mali at 12 months’ time (Zambrana et al., 2019). Another study by Sheflin et al. (2015) showed a significant increase in the abundance of *Methanobrevibacter smithii, Paraprevotella clara, Ruminococcus flavefaciens, Dialister succinatiphilus, Bifidobacterium* sp., *Clostridium glycolicum, Barnesiella intestinihominis, Anaerostipes caccae* and *Ruminococcus bromii* OTUs after heat stabilized rice bran was fed to people (3 g/day) (Sheflin et al., 2015). The differences in the enriched taxa could be because of the unique inoculum used in this study. However, both of the above studies had lower resolution and reported the enrichment of different taxa which could be attributed to a different variety of rice bran being used and individualized host factors (Stewart et al., 2005; Ridaura et al., 2013; Goodrich et al., 2014). The compounding host factors along with variation in age and geography (Yatsunenko et al., 2012), diet pattern (Wu et al., 2011), lifestyle (Benedict et al., 2016; Cook et al., 2016; Karl et al., 2017), etc., play a crucial role in determining the gut microbiota composition, thus masking the actual effect of the substrate alone on the microbiome. In contrast, this study supplies species-level resolution eliminating host interference and shows *Veillonella Prevotella, Dialister, Bacteroides* and *Alistipes* species are significantly enriched while *Oscillibacter, Eubacterium* and *Citrobacter* species are significantly reduced (Figure 3).

Similar to rice bran, previous studies analyzing microbial composition after quercetin supplementation yielded variable results among different hosts. *Enterobacteriaceae* and *Fusobacteriaceae* were reported to be positively related to quercetin supplementation whereas *Sutterellaceae* and *Oscillospiraceae* were found to be negatively correlated (Tamura et al., 2017). However, Lin et al. (2019) reported an increase in abundances of *Bifidobacterium, Bacteroides, Lactobacillus* and *Clostridium*, with a reduction of *Fusobacterium* and *Enterococcus* in mice fed with quercetin (Lin et al., 2019). With higher resolution and removal of host factors, our results contrast with both studies and show enrichment of *Acidaminococcus intestini* and decreased abundances of *E. limosum, E. faecalis, A. caccae, D. longicatena* and *C. catus*. The domination of *Enterobacteriaceae* in medium supplemented with quercetin (Figure 2C) might have resulted in lower enrichment of the bacterial taxa as *Enterobacteriaceae* has been reported to affect quercetin metabolism by directly or indirectly inhibiting quercetin degrading bacteria (Tamura et al., 2017).

When the combined effect of quercetin and rice bran were analyzed, we find that majority of the microbial shift is due to the supplementation of rice bran (Figure 2 and Supplementary Figures 2, 3). Most of the taxa enriched or decreased in the combination were very similar to those affected by rice bran supplementation alone. Compared to quercetin and rice bran supplementation separately, the combination was observed to significantly reduce members of the *Enterobacteriaceae* family (*Escherichia* and *Citrobacter* sp.) (Supplementary Table 1 and Figure 5). *Enterobacteriaceae* consists of class of pathogens that are low in abundance but have potential to grow and dominate during dysbiotic conditions (Seksik et al., 2003; Walker et al., 2011; Taur et al., 2012). The reduction of *Enterobacteriaceae* by combined quercetin and rice bran suggests a possibly beneficial effect on the host.

The reduction of *Enterobacteriaceae* members was highly correlated with greater propionate levels in quercetin and rice bran combined medium (Figures 4G, 5). Propionate, along with other SCFAs produced by the gut microbiota, has been previously implicated in regulating the intestinal morphology and functions (Scheppach, 1994). Interestingly, unlike butyrate that is used as an energy source for the colonocytes, the health benefits of propionate are not restricted to the colon. Propionate has been shown to decrease liver lipogenesis, and hepatic and plasma cholesterol levels in rats. It has also been implicated in reducing obesity, by stimulating satiety in mice models. Importantly, propionate has also been demonstrated as a potential anti-inflammatory and anti-cancer agent. The reported anti-inflammatory abilities of propionate are suggested to occur through the inhibition of Nuclear Factor-Kappa B and suppression of IL-6 mRNA and other immune-related gene expression, while its anti-cancer effects are thought to occur through inhibition of Histone Deacetylases (HDACs) and regulation of the AP-1 pathway (Tedelind et al., 2007; Hosseini et al., 2011; Nepelska et al., 2012). Therefore, our observations of increased propionate levels, along with previous reports on the health benefits of propionate, demonstrates the importance of both flavonoids and fiber in the diet. This study, for the first time, reports the combined effect of quercetin and rice bran on the gut microbial composition in the absence of interfering host factors. Here, we report that the gut microbial composition was altered favorably by quercetin and rice bran to result in a significant reduction of opportunistic pathogens that could potentially provide additional health benefits to the host.

## Data Availability Statement

The raw sequences are deposited in NCBI under BioProject PRJNA606575.

## Ethics Statement

The studies involving human participants were reviewed and approved by Institutional Review Board, South Dakota State University. The patients/participants provided their written informed consent to participate in this study.

## Author Contributions

JS and ER conceived and designed the manuscript. SG, SW, and RS performed the experiments. SG analyzed the data and wrote the first draft with input from all authors. JS, ER, and GB acquired the resources and supervised the manuscript. All authors contributed to the article and approved the submitted version.

## Conflict of Interest

The authors declare that the research was conducted in the absence of any commercial or financial relationships that could be construed as a potential conflict of interest.

## Publisher’s Note

All claims expressed in this article are solely those of the authors and do not necessarily represent those of their affiliated organizations, or those of the publisher, the editors and the reviewers. Any product that may be evaluated in this article, or claim that may be made by its manufacturer, is not guaranteed or endorsed by the publisher.

## Footnotes

1
https://www.bioinformatics.babraham.ac.uk/projects/fastqc/

2
https://software.broadinstitute.org/morpheus/

## Supplementary Material

Supplementary Figure 1 Exploratory analysis of predicted taxa and alpha diversity of microbiota in various treatments **(A)** Rarefaction curves measuring the bacterial diversity in fecal communities. The curves are based on V3-V4 16S rRNA gene sequences obtained from a total of 48 samples (12 each from control, QC, RB, and QC + RB) from day 4, 7, 14, and 21 and duplicate inoculums **(B)** Alpha diversity measure (Shannon diversity) between Control, supplemented with QC, supplemented with RB and supplemented with QC + RB determined using shotgun metagenome sequence analysis at day 21. Kruskal–Wallis test was performed for the Shannon diversity indices obtained for the groups (*p* = 0.27).

Supplementary Figure 2 Beta diversity assessment of the microbiota communities formed at days 4, 7, 14, and 21 among four groups of media condition (Control, QC, RB, and QC + RB) determined using 16S rRNA sequence analysis. The communities were ordinated using the “MDS” method followed by Bray-Curtis distance calculation to visualize the difference between the groups. Statistically, PERMANOVA using adonis was calculated on the beta diversity with *p* = 0.004. The upper panel represents the beta diversity in early stages (day 4 and day 7) and the lower panel represents in later stages (day 14 and day 21).

Supplementary Figure 3 Impact of quercetin (QC) and rice bran (RB) supplementation on bacterial taxonomy. Species-wise variation of the most abundant (top 25) of the bacterial taxa determined by shotgun metagenome sequencing at day 21 (endpoint) for control, QC, RB, and QC + RB conditions in triplicate.

Supplementary Figure 4 Heatmap depicting the functional pathways in the metagenomes at day 21 obtained from shotgun metagenomic sequencing. KEGG modules obtained from GHOSTKOALA for each metagenomic sequence was plotted as either complete, 1 block missing, 2 blocks missing or incomplete.

Supplementary Figure 5 Biplot showing ordination of short-chain fatty acid production and bacterial communities formed at day 21 in control, QC, RB, and QC + RB conditions. Abundances of the bacterial communities at day 21 determined by shotgun metagenomics were used with SCFAs profiles of day 21 to generate the plot.

Supplementary Table 1 List of all differentially abundant taxa in medium with quercetin (QC), medium with rice bran (RB) and medium with both quercetin and rice bran (QC + RB) compared to control. DESEq2 was used in shotgun metagenome sequences on day 21 for identification of differentially present taxa in the quercetin and rice bran supplemented media conditions when compared to control.
