# Supplementary figures and images for: Positive Synergistic Effects of Quercetin and Rice Bran on Human Gut Microbiota Reduces Enterobacteriaceae Family Abundance and Elevates Propionate in a Bioreactor Model
Source: Front Microbiol. 2021 Sep 30;12:751225. doi: 10.3389/fmicb.2021.751225 (PMC8516403; doi:10.3389/fmicb.2021.751225)

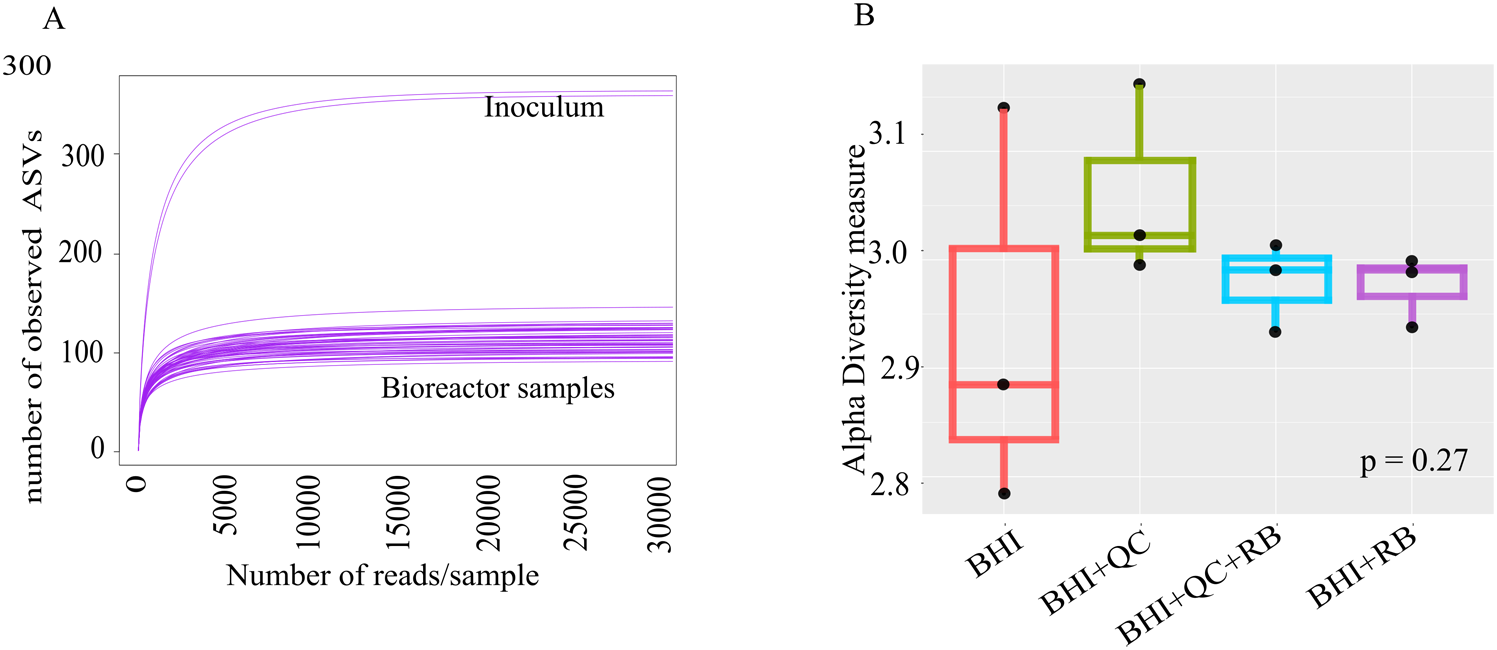

Supplement: Supplementary Figure 1 — Exploratory analysis of predicted taxa and alpha diversity of microbiota in various treatments (A) Rarefaction curves measuring the bacterial diversity in fecal communities. The curves are based on V3-V4 16S rRNA gene sequences obtained from a total of 48 samples (12 each from control, QC, RB, and QC + RB) from day 4, 7, 14, and 21 and duplicate inoculums (B) Alpha diversity measure (Shannon diversity) between Control, supplemented with QC, supplemented with RB and supplemented with QC + RB determined using shotgun metagenome sequence analysis at day 21. Kruskal–Wallis test was performed for the Shannon diversity indices obtained for the groups (p = 0.27). [file Image_1.TIF]

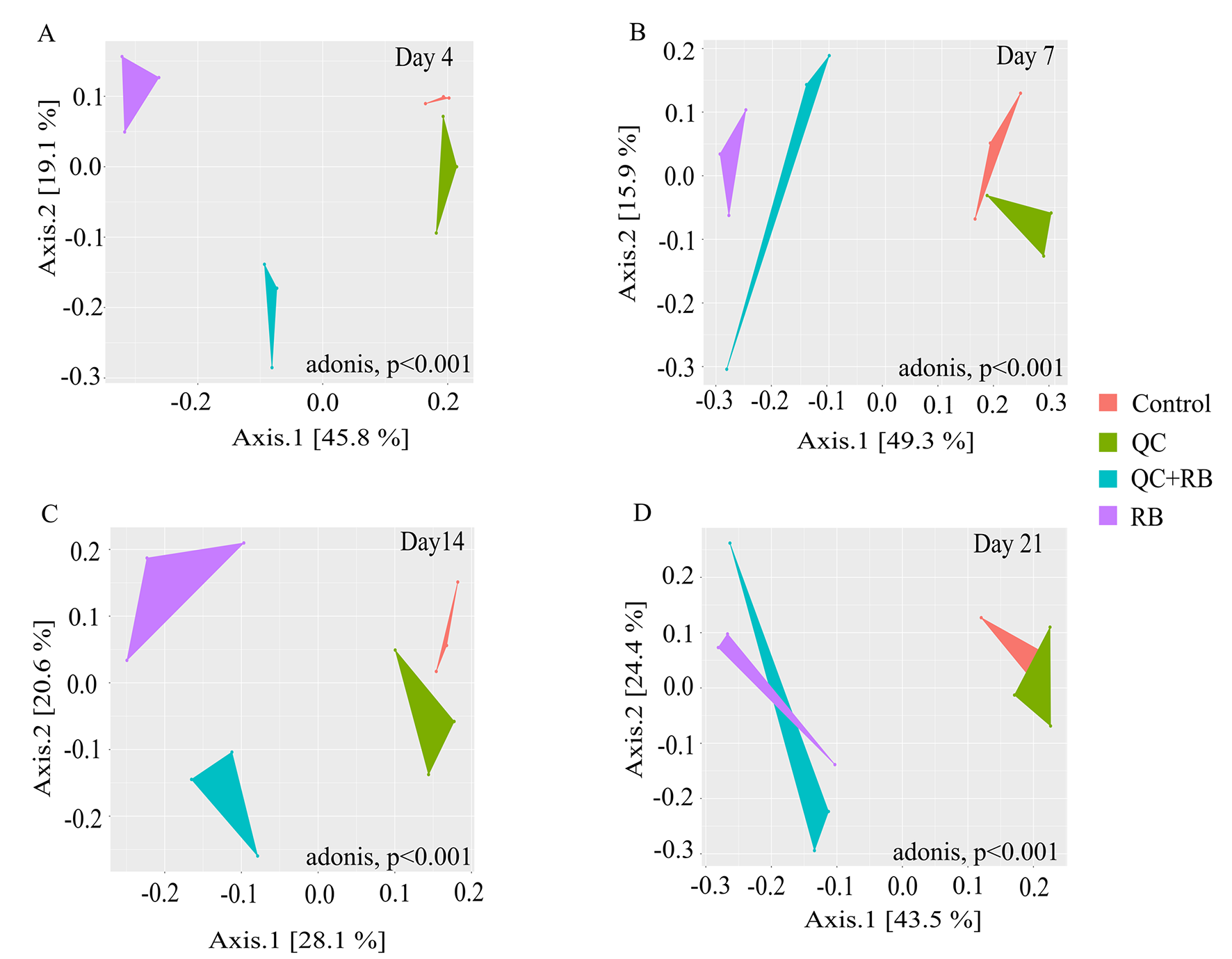

Supplement: Supplementary Figure 2 — Beta diversity assessment of the microbiota communities formed at days 4, 7, 14, and 21 among four groups of media condition (Control, QC, RB, and QC + RB) determined using 16S rRNA sequence analysis. The communities were ordinated using the “MDS” method followed by Bray-Curtis distance calculation to visualize the difference between the groups. Statistically, PERMANOVA using adonis was calculated on the beta diversity with p = 0.004. The upper panel represents the beta diversity in early stages (day 4 and day 7) and the lower panel represents in later stages (day 14 and day 21). [file Image_2.TIF]

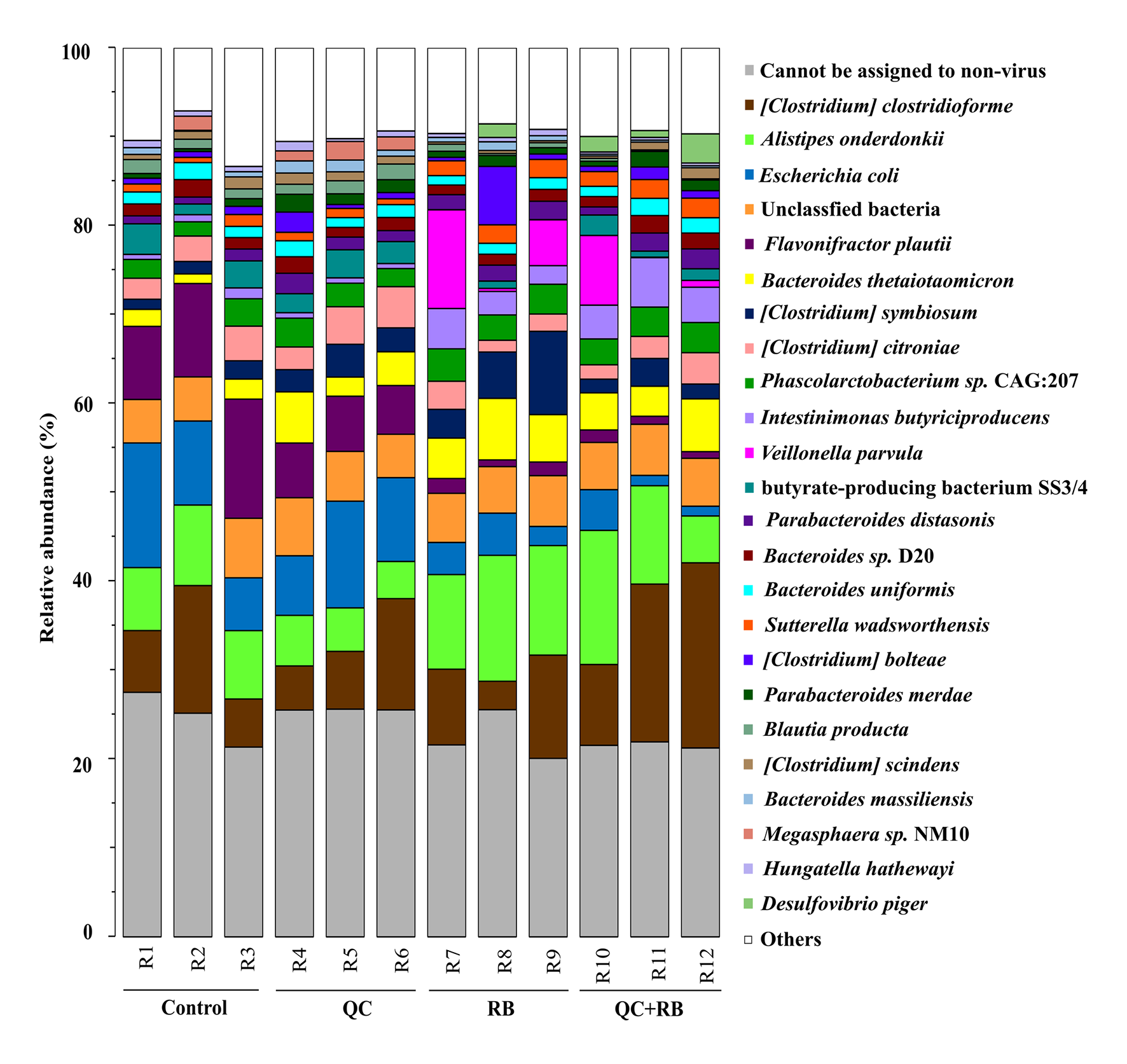

Supplement: Supplementary Figure 3 — Impact of quercetin (QC) and rice bran (RB) supplementation on bacterial taxonomy. Species-wise variation of the most abundant (top 25) of the bacterial taxa determined by shotgun metagenome sequencing at day 21 (endpoint) for control, QC, RB, and QC + RB conditions in triplicate. [file Image_3.TIF]

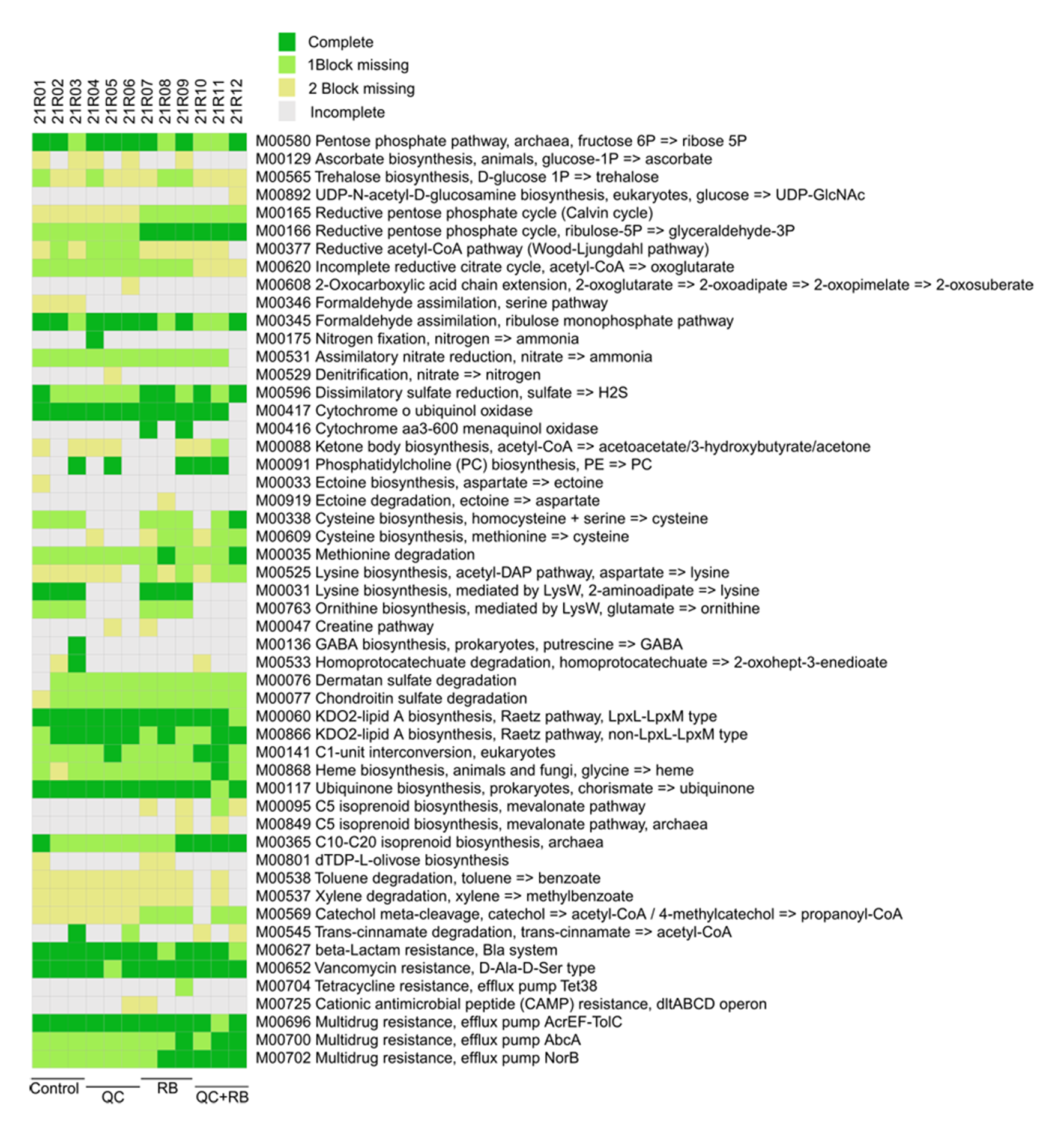

Supplement: Supplementary Figure 4 — Heatmap depicting the functional pathways in the metagenomes at day 21 obtained from shotgun metagenomic sequencing. KEGG modules obtained from GHOSTKOALA for each metagenomic sequence was plotted as either complete, 1 block missing, 2 blocks missing or incomplete. [file Image_4.TIF]

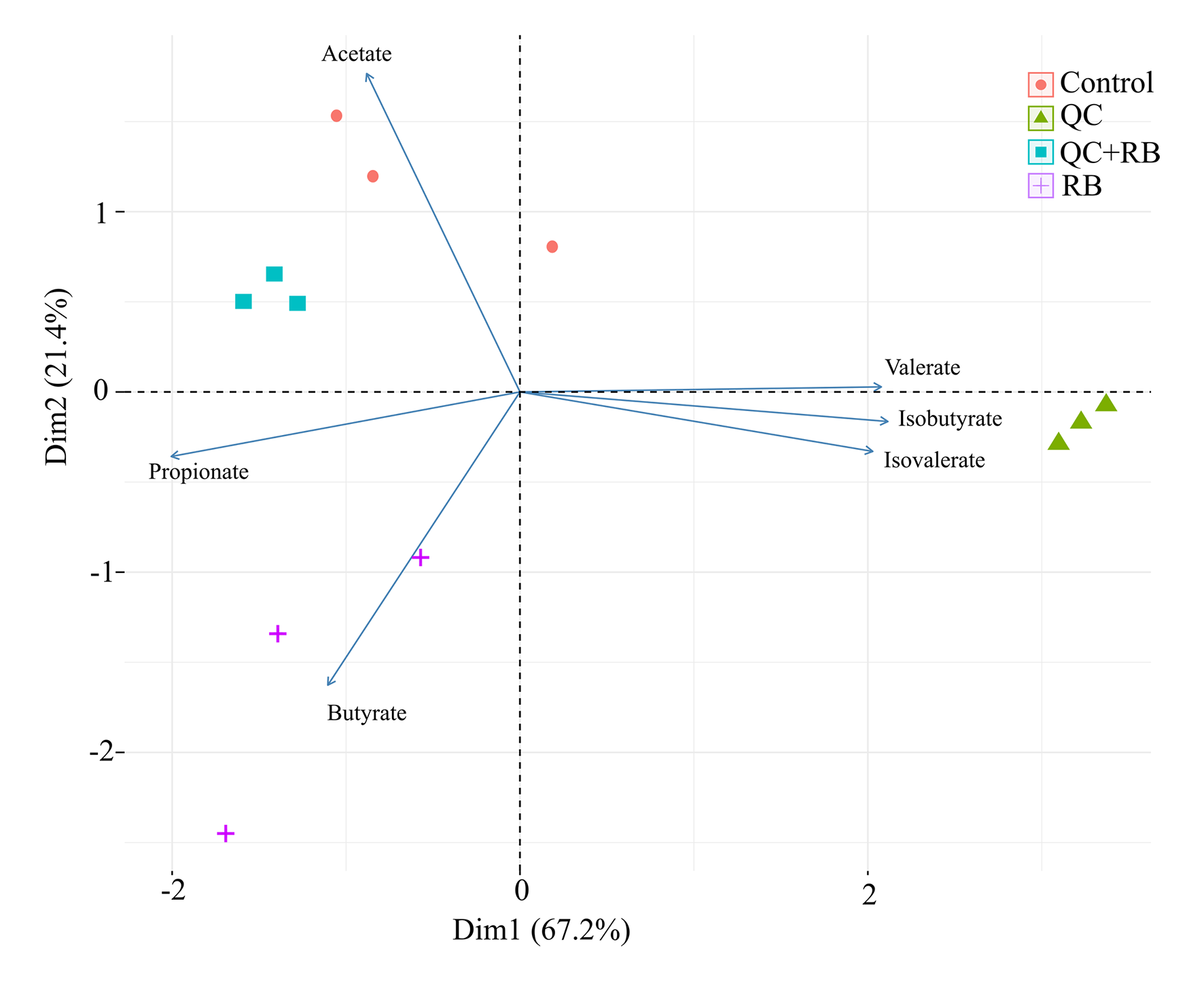

Supplement: Supplementary Figure 5 — Biplot showing ordination of short-chain fatty acid production and bacterial communities formed at day 21 in control, QC, RB, and QC + RB conditions. Abundances of the bacterial communities at day 21 determined by shotgun metagenomics were used with SCFAs profiles of day 21 to generate the plot. [file Image_5.TIF]
